# Non-parallel changes in songbird migration timing are not explained by changes in stopover duration

**DOI:** 10.7717/peerj.8975

**Published:** 2020-05-19

**Authors:** Nicholas N. Dorian, Trevor L. Lloyd-Evans, J. Michael Reed

**Affiliations:** 1 Department of Biology, Tufts University, Medford, MA, USA; 2 Manomet Inc., Manomet, MA, USA

**Keywords:** Mark-recapture, Cormack–Jolly–Seber, Bird banding, Hierarchical models, Quantile regresision, Stopover duration, Phenology

## Abstract

Shifts in the timing of animal migration are widespread and well-documented; however, the mechanism underlying these changes is largely unknown. In this study, we test the hypothesis that systematic changes in stopover duration—the time that individuals spend resting and refueling at a site—are driving shifts in songbird migration timing. Specifically, we predicted that increases in stopover duration at our study site could generate increases in passage duration—the number of days that a study site is occupied by a particular species—by changing the temporal breadth of observations and vise versa. We analyzed an uninterrupted 46-year bird banding dataset from Massachusetts, USA using quantile regression, which allowed us to detect changes in early-and late-arriving birds, as well as changes in passage duration. We found that median spring migration had advanced by 1.04 days per decade; that these advances had strengthened over the last 13 years; and that early-and late-arriving birds were advancing in parallel, leading to negligible changes in the duration of spring passage at our site (+0.07 days per decade). In contrast, changes in fall migration were less consistent. Across species, we found that median fall migration had delayed by 0.80 days per decade, and that changes were stronger in late-arriving birds, leading to an average increase in passage duration of 0.45 days per decade. Trends in stopover duration, however, were weak and negative and, as a result, could not explain any changes in passage duration. We discuss, and provide some evidence, that changes in population age-structure, cryptic geographic variation, or shifts in resource availability are consistent with increases in fall passage duration. Moreover, we demonstrate the importance of evaluating changes across the entire phenological distribution, rather than just the mean, and stress this as an important consideration for future studies.

## Introduction

The timing of animal migration—the seasonal movements between breeding and non-breeding sites—depends on reliable biotic and abiotic cues, such as food availability and temperature (Dingle, 1996). Yet, many of these cues have changed in magnitude, timing and frequency over the last century due to climate change and anthropogenic development (Newson et al., 2009). As a result, shifts in the timing of animal migration have occurred across taxa (Walther et al., 2002). For example, beluga whales now postpone their autumn migration in response to delayed sea ice formation (Hauser et al., 2017), and potato leafhoppers have advanced their northbound migration due to earlier springtime (Baker, Venugopal & Lamp, 2015). Although these shifts can be inconsequential when animals track their resources (Bartomeus et al., 2011), they can also lead to decreased fitness when animals become mismatched in space and time with their resources (Reed, Jenouvrier & Visser, 2012; Mayor et al., 2017; Kharouba et al., 2018). To date, far more studies have focused on documenting patterns in phenology, that is, whether or not shifts have occurred (Butler, 2003; Marra et al., 2005; Visser & Both, 2005; Otero et al., 2014; Usui, Butchart & Phillimore, 2017; Stepanian & Wainwright, 2018), rather than how shifts are occurring (Gill et al., 2014; Kharouba et al., 2018; Schmaljohann, 2019). Information on phenological patterns and the mechanisms driving them is needed to understand the relationship between migration and environmental change and to develop effective conservation guidelines for imperiled species (Bowlin et al., 2010; Allen & Singh, 2016; Guérin et al., 2017).

To describe patterns in migration phenology, many studies use either changes in the date of first occurrence or in the mean date of arrival at study site (Butler, 2003; Tryjanowski, Kuźniak & Sparks, 2005; Miller-Rushing et al., 2008; DeLeon, DeLeon & Rising, 2011). Using first occurrence is problematic since it is sensitive to changes in population size (Tryjanowski, Kuźniak & Sparks, 2005). Analyses of change in mean date are also limited, although perhaps less obviously so, in that they assume that phenological responses are parallel across the entire distribution of activity, such that the earliest arriving individuals are changing their timing at the same rate as the latest-arriving individuals. However, phenological reaction norms are known to vary among individuals and environmental conditions vary throughout the migration season (Battley, 2006; Inouye, Ehrlén & Underwood, 2019; Senner et al., 2019). Therefore, non-parallel responses in migration timing must be considered.

**Figure 1:**
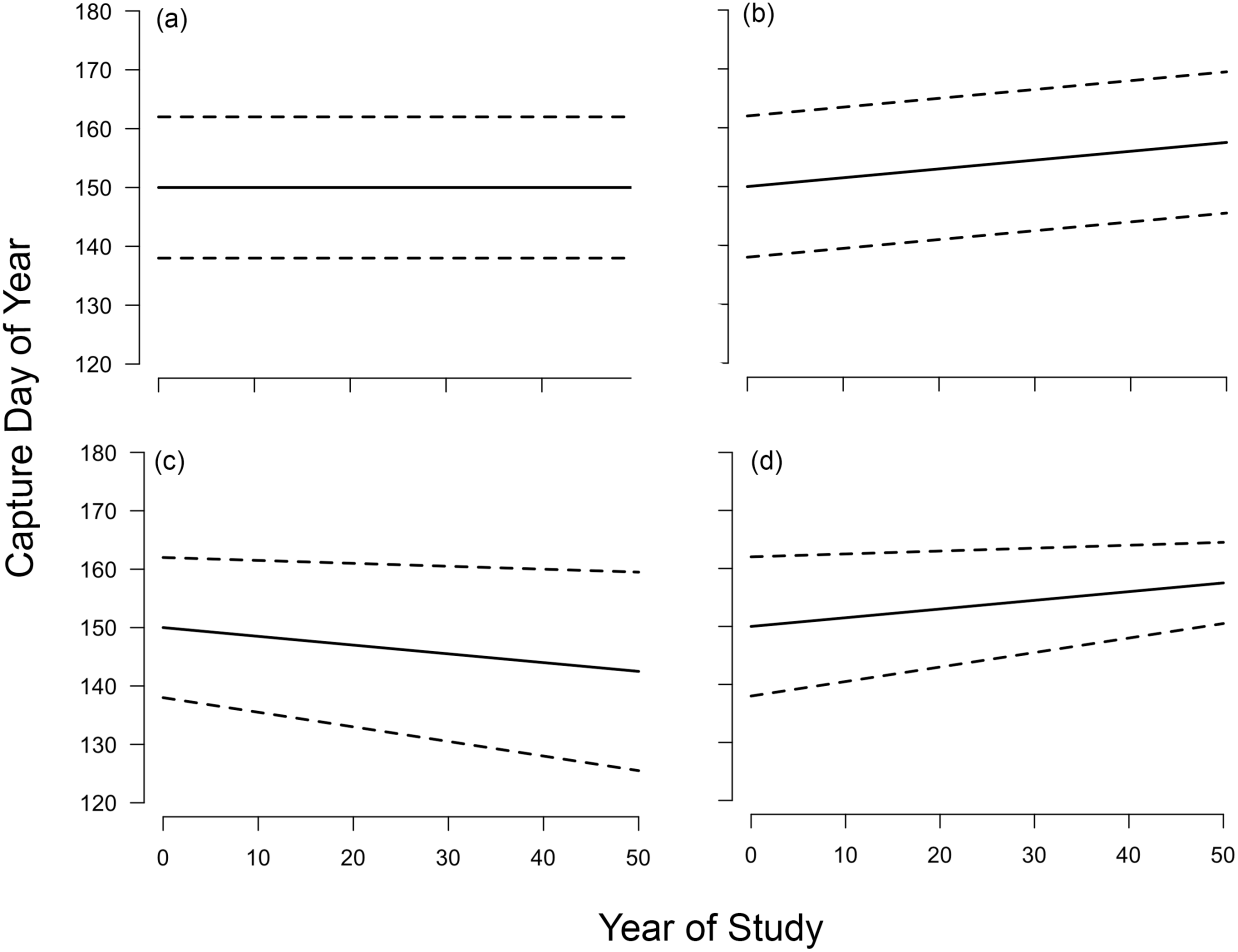
Possible long-term changes in timing and passage duration at a migratory stopover site. Dotted lines indicate 0.15 and 0.85 quantiles, respectively, and solid line represents median. (A) No change in phenology, that is, quantile slopes not significantly different from zero; (B) parallel change in phenology across quantiles (C) increase in passage duration, that is, non-parallel response with diverging lower and upper quantile slopes; (D) decrease in passage duration, that is, non-parallel response with converging lower and upper quantile slopes. Scenarios (A) and (B) lead to no overall change in passage duration. We hypothesized that increases and decreases in stopover duration over our study period would be able to explain changes in passage duration outlined in (C) and (D), respectively. Figure redrawn from Barton & Sandercock (2018).

In this context, a non-parallel response means that the earliest-timed individuals are shifting their migration timing differently than are the latest-timed individuals (Lehikoinen et al., 2019; Fig. 1). Non-parallel responses result in a change in the temporal breadth of observations over time, which in the case of migration timing, can either increase or decrease the period of time that a study site is occupied by a particular species (hereafter, passage duration; Fig. 1). Though underappreciated, the existence of non-parallel responses underscores that using just one metric to describe phenological changes, such as the mean arrival date at a study site, oversimplifies how migratory populations respond to environmental change.

Changes in mean songbird migration timing are well documented, but we know much less about whether these changes in migration timing are parallel or non-parallel across the distribution of arrival dates at a stopover site. Strong advances in mean arrival date have been documented consistently in spring (Mills, 2005; Marra et al., 2005; Gordo & Sanz, 2006; Jonzén et al., 2006; Miller-Rushing et al., 2008; Van Buskirk, Mulvihill & Leberman, 2009), whereas advances, delays, and no change in mean fall timing have been found (Jenni & Kéry, 2003; Smith & Paton, 2011; Kovács et al., 2012; Ellwood et al., 2015; Barton & Sandercock, 2018). Fewer studies have compared changes in the mean to changes in the timing of early-and late-arriving birds across seasons. Although parallel shifts are sometimes found (e.g., pied flycatcher *Ficedula hypoleuca*; Cadahía et al., 2017), more studies find evidence of non-parallel responses. Advances in spring are often strongest in early-arriving individuals across diverse songbird taxa, leading to increases in passage duration (Van Buskirk, Mulvihill & Leberman, 2009; Lehikoinen et al., 2019; Covino, Horton & Morris, 2020), but one study found that delays in both early- and late-arriving individuals decreased spring passage duration for pacific-slope flycatcher (*Empidonax difficilis*) and Wilson’s warbler (*Cardellina pusilla*; Barton & Sandercock, 2018). In fall, one study found advances in early-arriving individuals but not in late-arriving individuals (Barton & Sandercock, 2018), whereas another found that delays occurred across the entire distribution, but were strongest in late-arriving birds (Kovács et al., 2012). These varied, non-parallel responses likely arose from factors that differentially affect early-and late-arriving birds, including spatiotemporal variation in weather, resources, and heterogeneity in phenological responses among individuals (Gordo & Sanz, 2006; Studds & Marra, 2011; Senner et al., 2019). To our knowledge, however, no studies have explicitly considered the factors that affect the magnitude and direction of non-parallel responses in migration timing.

One metric that integrates the numerous and complex effects of these factors is stopover duration: the time that individuals spend resting and refueling at a site during their journey (Newton, 2008). As such, we hypothesize that population-level changes in stopover duration could lead to changes in observed passage duration. This hypothesis is testable because of the way migratory passerines are typically studied—standardized capture using mist-netting—which samples a fraction of the population of migrants passing through a site on each day of the season across many years (Ralph & Dunn, 2004). Since the exact date on which a bird arrived at a study site is unknown, a longer stopover duration among all individuals of a species would increase the chance that an individual from the population is observed at least once during its stay. Thus, given a systematic increase in population-level stopover duration at a study site, we would expect an increase in the observed passage duration at the same site and vise versa.

In this article, we used a 46-year dataset of systematic passerine migration monitoring from coastal MA (USA) to test the hypothesis that changes in stopover duration are sufficient to generate changes in songbird passage duration in both spring and fall. First, we described changes in migration timing, including passage duration, across the distribution of arrival dates using quantile regression (Cade & Noon, 2003). Following Barton & Sandercock (2018), we acknowledged four possible outcomes: no shifts throughout the distribution; parallel shifts in phenology between the earliest and latest arriving individuals; increase in passage duration in which the tails of the distribution are changing in opposite directions; and decrease in passage duration (Fig. 1). We contextualized our findings across migration distances (short, medium and long) and seasons (spring and fall). Then, we asked whether changes in stopover duration were sufficient to generate shifts in passage duration. We estimated change in stopover duration for each species and asked whether these changes were correlated with the observed phenological shifts. If changes in stopover duration generate non-parallel responses in migration phenology, then a change in passage duration would be accompanied by a change in stopover duration of a similar magnitude and direction. Since most passerines do not have stopover site fidelity (Catry et al., 2004) or make stopovers of the same length across years (Morris et al., 2006; Hochachka & Fiedler, 2008; Calvert et al., 2009), we only correlate changes in stopover duration with shifts in passage duration at Manomet.

## Materials and Methods

### Bird banding

Migrant birds passing through Manomet in Plymouth, MA, USA (41.920°N, 70.543°W) were banded every spring (15 April to 15 June) and fall (15 August to 15 November) from 1970 to 2015. Between 45 and 50 nylon mist-nets (12 m long, 2.6 m high, four panels, 36 mm extended mesh) were positioned throughout Manomet’s peninsular property on the western edge of Cape Cod Bay. The locations of mist-nets were essentially unchanged over all years to avoid bias in capture rates. Mist-nets were operated five to seven days per week each season, weather permitting, from 30 min before sunrise to 30 min after sunset. Each unbanded bird captured was fitted with a uniquely numbered aluminum band. All captured birds were identified to species and the time and date of collection were recorded.

Banding each year was conducted at Manomet under supervision of TLE who was in possession of an active Federal Bird Banding permit from the US Geological Survey Banding Lab (#09859) and a bird banding permit from the Massachusetts Division of Fish and Wildlife (#022.19BB).

We selected study species according to the following criteria: (1) a migratory passerine that (2) does not breed at the site, (3) does not winter at the site in significant numbers, with (4) adequate capture and recapture data throughout all years of the study (>300 individuals per season), and that (5) can be classified as a short-, medium-, or long-distance migrant based on the published great circle distance between the centroids of its breeding and wintering grounds (La Sorte et al., 2015). This led us to choose 11 species to investigate: four long-distance migrants, three medium distance migrants, and four short-distance migrants for investigation. Scientific names and migration distances of study species are listed in Table 1.

**Table 1:** Migration distance and phenology of eleven passerine species passing through Manomet in MA, USA from 1970–2015. Migration distance classification was determined based on the great circle distance between the centroid of the breeding and wintering grounds for each species, after La Sorte et al. (2015). For each season, passage durations are the differences in days between the 0.85 quantile and 0.15 quantile. Sample sizes presented for each season were the same for both quantile regression and stopover duration analysis.

Species	Scientific name	Migration distance	Median spring arrival	Spring passage duration	*n*	Median fall arrival	Fall passage duration	*n*
Veery	*Catharus fuscescens*	Long	May 20	14	610	September 12	20	542
Swainson’s Thrush	*Catharus ustulatus*	Long	May 26	12	1,505	September 24	25	1,233
Blackpoll Warbler	*Setophaga striata*	Long	May 28	16	1,101	September 27	25	7,512
Canada Warbler	*Cardellina canadensis*	Long	May 28	11	1,452	August 30	25	467
Ovenbird	*Seiurus aurocapilla*	Medium	May 18	13	1,488	September 6	36	651
Black-and-white Warbler	*Mniotilta varia*	Medium	May 23	14	3,351	September 18	35	793
Magnolia Warbler	*Setophaga magnolia*	Medium	May 15	15	2,423	September 6	27	1,079
Hermit Thrush	*Catharus guttatus*	Short	April 30	18	1,540	October 21	22	1,929
Swamp Sparrow	*Melospiza georgiana*	Short	May 12	22	1,327	October 12	22	1,126
Brown Creeper	*Certhia americana*	Short	April 24	14	187	October 11	25	1,587
Ruby-crowned Kinglet	*Regulus calendula*	Short	April 30	18	1,235	October 15	21	1,848

For each species, we divided the data into two seasons (spring and fall) across all years (1970–2015). We converted capture dates to day-of-year and rounded the time of capture to the nearest hour. For birds that were captured twice in one hour, only the earlier record was retained. The few individuals that overwintered at the site were removed from analyses (<1 hermit thrush, ruby-crowned kinglet, and swamp sparrow per year). We summarize the migration distance and general migration phenology of each species in Table 1. We also test for differences in mean passage duration and stopover duration among seasons and migration distances using type II ANOVA tests fit via the *car* package in R (Fox & Weisberg, 2010).

### Measuring phenological change

We assessed changes over time in migration timing using quantile regression. Instead of estimating the conditional mean like conventional linear regression, quantile regression evaluates one or many conditional quantiles of the data (Cade & Noon, 2003). Quantile regression is a powerful—albeit underused—tool for studying changes in phenology because it can reveal whether there are differences in the rates of change in the timing of events across the entire distribution. Furthermore, unlike estimations based on first arrival date, quantile regression is robust to changes in variance, such as population declines, across the range of the predictor (Cade & Noon, 2003).

We estimated the changes in arrival date for three quantiles: 0.15 (early arrivals), 0.50 (median arrivals), 0.85 (late arrivals). Positive quantile slopes indicate delays in migration timing, whereas negative slopes indicate advances. We also calculated the difference between the slopes of the 0.85 and 0.15 quantiles, which measures the rate of change in passage duration and can be interpreted as degree of non-parallel response; a significantly positive value indicates an increase in the passage duration, whereas a negative value indicates a decrease (Fig. 1). For the slope of each quantile and our derived metric, we used non-parametric bootstrapping over 1,000 replicates to estimate 95% confidence intervals. We assigned significance if the confidence intervals did not overlap zero. We report slopes and standard errors in days per decade. Truncated versions of our dataset from Manomet have been analyzed before for changes in mean arrival date in both spring (33 years; Miller-Rushing et al., 2008) and fall (43 years; Ellwood et al., 2015), so we also compare our findings to these past results.

### Estimating stopover duration

We estimated stopover duration using Cormack–Jolly–Seber open population (CJS) models from mark-recapture data gathered via mist nets (Schaub et al., 2001). To do so, we first built capture histories of all individuals for each species-season combination. Each capture history is a binary sequence denoting captured/not captured for each bird on each day that banding took place. When collated across individuals, capture histories form a data matrix of *N* independent individuals (rows) surveyed over *k* occasions (columns). In our case, *N* is the total number of unique birds captured of a single species within a season and *k* is the standardized length of the banding season (spring = 62 days, fall = 93 days). There were not sufficient brown creeper captures in spring to conduct mark-recapture analyses, so we excluded that dataset. We did not include age or sex as covariates in any of our models.

Then, we estimated stopover duration using CJS models fit in a Bayesian state-space framework. CJS models jointly estimate two parameters from mark-recapture data—the probabilities of survival (φ) and recapture (*p*) (Schaub et al., 2001; Morris et al., 2006; Schaub, Jenni & Bairlein, 2008). Assuming negligible mortality of individuals during the sampling period, stopover duration (i.e., length of stay after first capture) can be calculated from survival probability by using the classic life-expectancy formula: –1/ln(φ) (Schaub et al., 2001). By simultaneously estimating a recapture probability, these models account for imperfect detection of individuals.

We fit a hierarchical group-effects model in order to account for heterogeneity in survival and recapture across all years (Kéry & Schaub, 2012; Lok et al., 2019). The benefit of applying hierarchical models to this dataset is that estimates are informed by underlying year-to-year variance, so that years with sparse data are informed by years with more data (Hochachka & Fiedler, 2008; Cressie et al., 2009; Kéry & Schaub, 2012). Compared to frequentist approaches, it is relatively straightforward to build mark-recapture models with random effects in a Bayesian framework (Kéry & Schaub, 2012; Halstead et al., 2012). In our model, we kept survival and recapture probabilities constant within each banding season, so that probabilities were equal on each day, but we allowed these parameters to vary hierarchically among years. In other words, we accounted for inter-annual, but not intra-annual variation in survival and recapture.

We fit each model to both seasons for all 11 species, except brown creeper for which spring data were insufficient, totaling 21 models. We used uninformative logit-scale priors Normal (0, 0.001) (parameterized as mean and tau = 1/variance) for both survival and recapture. Logit-scale priors ensure that probabilities are bounded between 0 and 1. We used a Uniform (0, 5) prior for the standard deviation on the logit scale of both random effects terms. Each model was run across three chains for 30,000 iterations each with a burn-in of 10,000, retaining a third of all iterations. We assessed convergence by visually inspecting trace plots and quantitatively via the Gelman-Rubin statistic (R-hat < 1.05) (Kéry & Schaub, 2012). All parameters successfully converged.

To determine trends in stopover duration over time, we regressed the transformed survival probability estimates against year and converted the slopes to units of days per decade for comparison with shifts in passage duration. Significance of the slope was determined using a type II analysis of variance implemented through the *car* package (Fox & Weisberg, 2010). Since our annual stopover estimates were made using a hierarchical model, they experience shrinkage towards the mean (Cressie et al., 2009). As a result, any trends in stopover duration over time are conservative. Non-hierarchical model alternatives, in which each year is fit as a separate fixed effect, would produce less conservative estimates, but they did not produce tractable parameter estimates for uncommonly captured species (e.g., veery) or those with strongly declining populations (e.g., blackpoll warbler, recent years had insufficient spring data, Supplemental Materials S4).

All analyses were conducted in R 3.5.1 (R Core Team, 2018). QR analyses were conducted using the *quantreg* package (Koenker, 2018). CJS models were fit using JAGS 4.3.0 (Plummer, 2003) and implemented via the R package jagsUI (Kellner, 2018). Code used to conduct QR and estimate stopover duration is available in Supplemental Materials.

### Comparison of phenology and stopover trends

We tested whether systematic changes in stopover duration were correlated with changes in the window of migratory bird arrival over 46 years. First, we expected a stopover slope > 0 to generate an increase in passage duration, a slope < 0 to generate a decrease, and a slope that is not significantly different from zero to generate no change. Second, we expected the magnitude of change in stopover duration to be less than or equal to the observed change in passage duration in all cases. If the sign and magnitude of change in stopover duration is similar to the change in passage duration, then the two metrics should be correlated along a 1:1 axis. If not, this implies that other factors were more important for generating shifts in passage duration of songbirds at Manomet.

## Results

Across forty-six years and eleven passerine species, we analyzed 16,219 records in spring and 18,767 records in fall of which 4,801 (29.6%) and 7,693 (41.0%) were recapture events, respectively. Sample sizes for all analyses are presented in Table 1.

Mean passage duration varied significantly across season and migration distance (Table 1; type II ANOVA season *F*(1, 16) = 78.18, *p* < 0.001; distance *F*(2, 16), *p* = 0.019; season × distance *F*(2, 16) = 1.99, *p* < 0.001). Across species, fall passage at Manomet lasted longer than spring migration (25.7 vs. 15.2 days). Short-distance migrants were observed for longer periods that were medium- or long-distance migrants (18.0 vs. 14.0 and 13.3 days, respectively; one-way ANOVA *F*(2,8) = 4.18, *p* = 0.06). Fall passage lasted significantly longer for medium-distance migrants than for long- or short-distance migrants (32.7 vs. 23.8 and 22.5 days, respectively; one-way ANOVA *F*(2, 8) = 10.56, *p* = 0.006). Average stopover duration was significantly shorter in spring than in fall (1.98 days vs. 3.34 days), but did not vary across migration distances (Table 2; ANOVA, season *F*(1, 15) = 15.24, *p* = 0.001; distance *F*(2, 15) = 0.94, *p* = 0.42; distance × season *F*(2, 15) = 1.74, *p* = 0.21).

**Table 2:** Changes in stopover duration for 11 passerine species over 46 years were weak and negative. Stopover duration estimates were made for each species in each season by fitting a hierarchical Cormack-Jolly-Seber mark-recapture model with a random effect of year, and trends were analyzed using standard linear regression on annual median estimates. Slopes and standard errors from linear regression on annual stopover duration estimates over time are reported in days per decade; significant effects (*p* < 0.05) are bolded. Sample sizes and scientific names are as reported in Table 1.

Species	Season	Median stopover duration (days)	Slope	SE	*p*-Value
Veery	Spring	1.55	−0.007	0.006	0.266
Swainson’s Thrush	Spring	1.14	−0.052	0.029	0.081
Blackpoll Warbler	Spring	1.18	−0.039	0.017	**0.026**
Canada Warbler	Spring	1.65	−0.001	0.015	0.932
Ovenbird	Spring	2.11	0.005	0.032	0.870
Black-and-white Warbler	Spring	2.39	−0.025	0.013	0.058
Magnolia Warbler	Spring	1.82	0.062	0.049	0.213
Hermit Thrush	Spring	2.97	−0.120	0.071	0.095
Swamp Sparrow	Spring	2.81	−0.026	0.017	0.120
Brown Creeper	Spring	INSUFFICIENT DATA			
Ruby-crowned Kinglet	Spring	2.20	0.000	0.009	0.968
Veery	Fall	3.77	−0.021	0.027	0.439
Swainson’s Thrush	Fall	3.49	−0.144	0.063	**0.027**
Blackpoll Warbler	Fall	2.95	−0.039	0.048	0.416
Canada Warbler	Fall	3.19	−0.024	0.018	0.190
Ovenbird	Fall	4.63	0.000	0.016	0.981
Black-and-white Warbler	Fall	3.63	−0.018	0.006	**0.004**
Magnolia Warbler	Fall	2.67	0.012	0.014	0.368
Hermit Thrush	Fall	4.44	−0.008	0.051	0.874
Swamp Sparrow	Fall	4.33	0.008	0.077	0.917
Brown Creeper	Fall	1.80	−0.015	0.016	0.352
Ruby-crowned Kinglet	Fall	1.93	0.064	0.040	0.122

**Figure 2:**
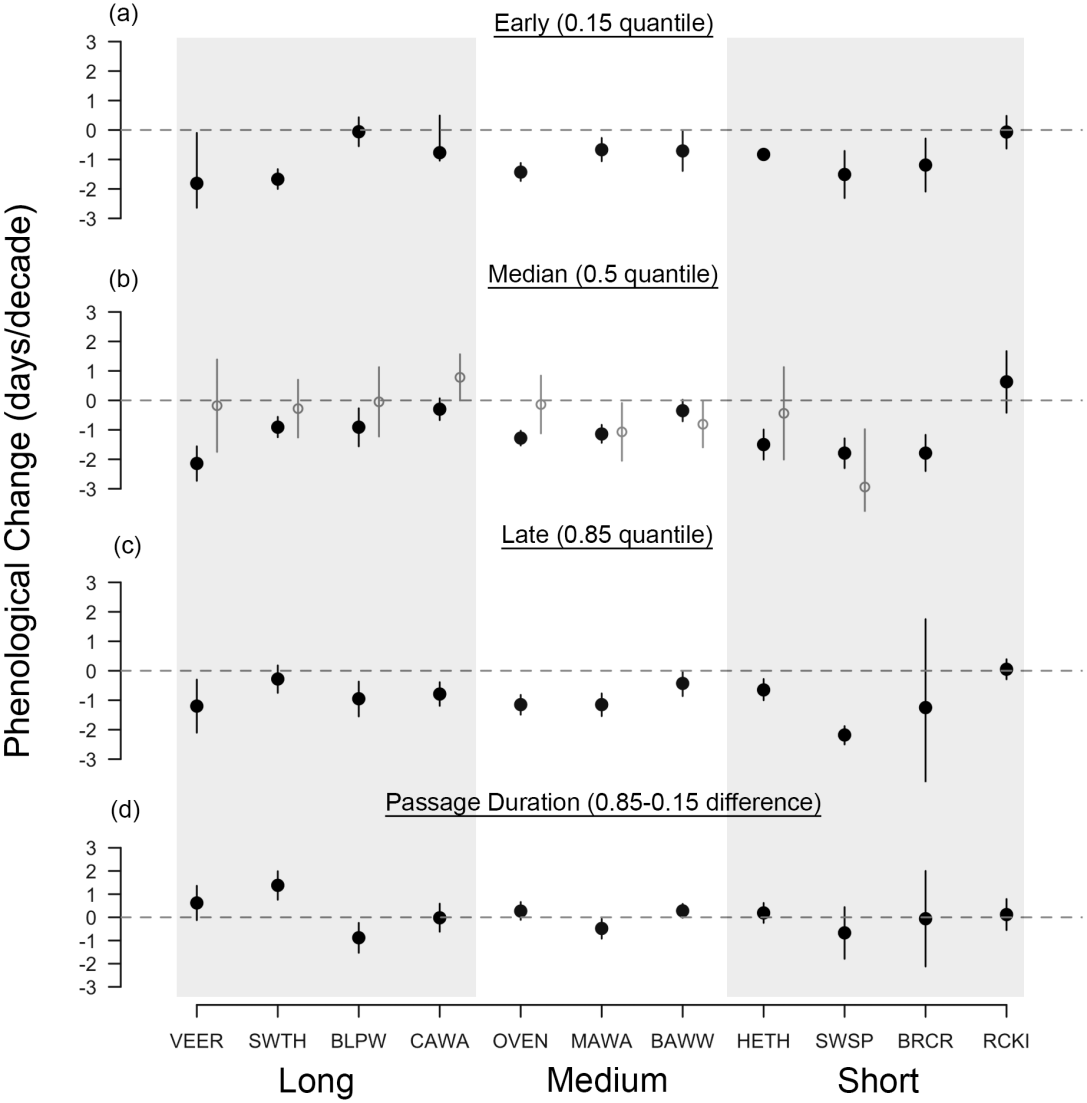
Long-term shifts in spring migration timing and passage duration of 11 passerine species from 1970 to 2015. Black points are quantile slopes ± 95% confidence intervals of changes in phenology across the distribution of arrival dates: (A) early arrivals (0.15 quantile), (B) median arrivals (0.5 quantile), (C) late arrivals (0.85 quantile) and (D) passage duration (0.85–0.15 quantile), a derived metric indicating the rate of change in the number of days that a species occurs at Manomet each season. For (D), positive values indicate an increase in the duration of migration at Manomet over the study period, whereas negative values indicate a decrease. Gray points indicate mean slopes ± 95% CI from a previous study that analyzed a truncated version of our spring dataset (1970–2002; Miller-Rushing et al., 2008). VEER (veery), SWTH (Swainson’s thrush), BLPW (blackpoll warbler), CAWA (canada warbler), OVEN (ovenbird), MAWA (magnolia warbler), BAWW (black-and-white warbler), HETH (hermit thrush), SWSP (swamp sparrow), BRCR (brown creeper), RCKI (ruby-crowned kinglet).

**Figure 3:**
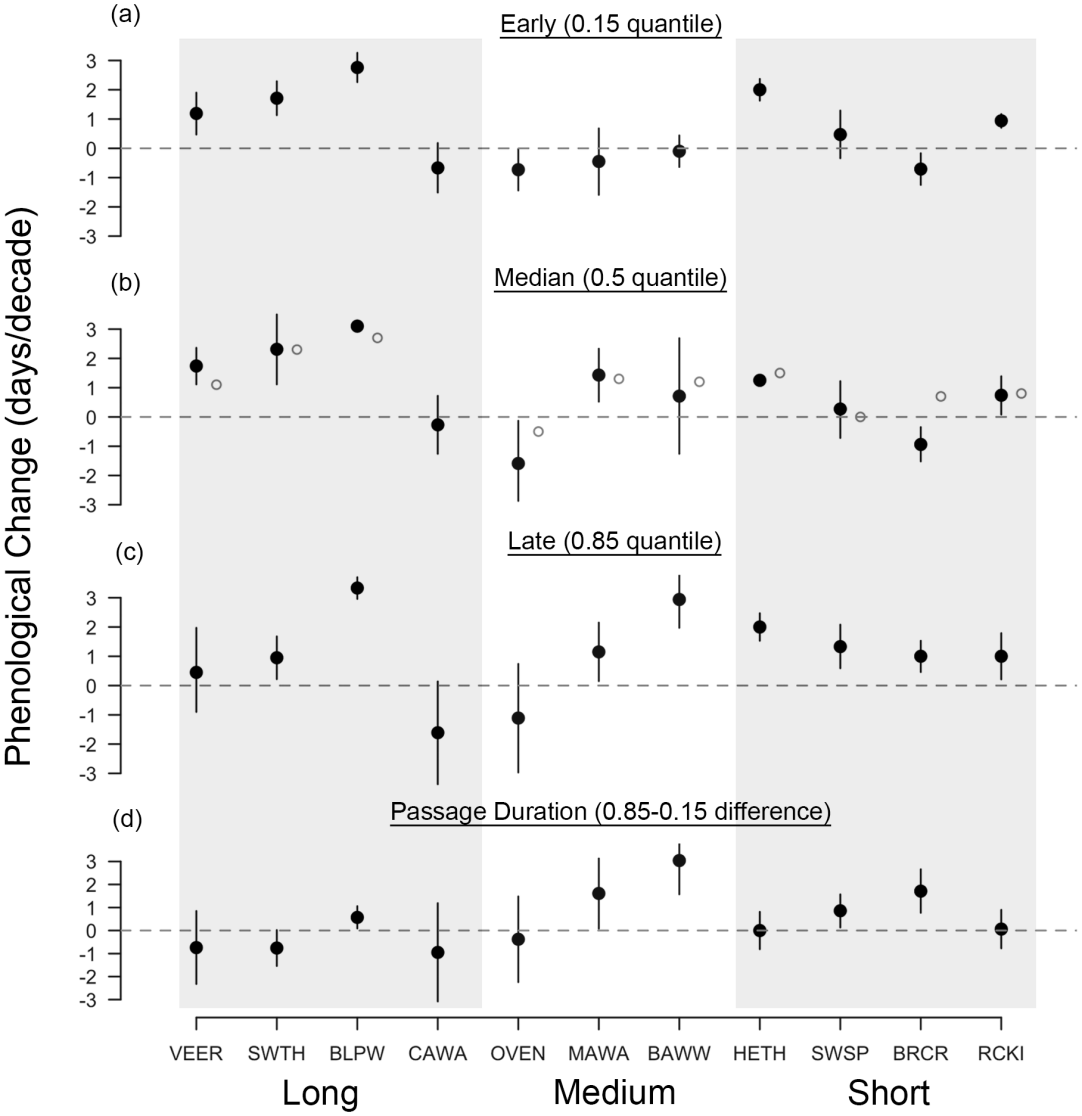
Long-term shifts in fall migration timing and passage duration of 11 passerine species from 1970 to 2015. Black points are quantile slopes ± 95% confidence intervals slopes of changes in phenology across the distribution of arrival dates: (A) early arrivals (0.15 quantile), (B) median arrivals (0.5 quantile), (C) late arrivals (0.85 quantile) and (D) passage duration (0.85–0.15 quantile), a derived metric indicating the rate of change in the number of days that a species occurs at Manomet each season. Interpretation of slopes is as explained in Fig. 2 caption. Gray points indicate mean slopes from a previous study that analyzed a truncated version of our fall dataset (1970–2012; Ellwood et al., 2015). VEER (veery), SWTH (Swainson’s thrush), BLPW (blackpoll warbler), CAWA (canada warbler), OVEN (ovenbird), MAWA (magnolia warbler), BAWW (black-and-white warbler), HETH (hermit thrush), SWSP (swamp sparrow), BRCR (brown creeper), RCKI (ruby-crowned kinglet).

Across all species, the median arrival date of passerines at Manomet was advancing in spring by 1.04 days per decade, regardless of migration distance (1.4 days per decade for the eight species significantly advancing; Fig. 2B). Changes in fall median arrival date were less consistent but averaged +0.80 days per decade across all species (Fig. 3B). The six birds that significantly delayed their arrival did so by an average of 1.8 days per decade (Fig. 3B).

**Figure 4:**
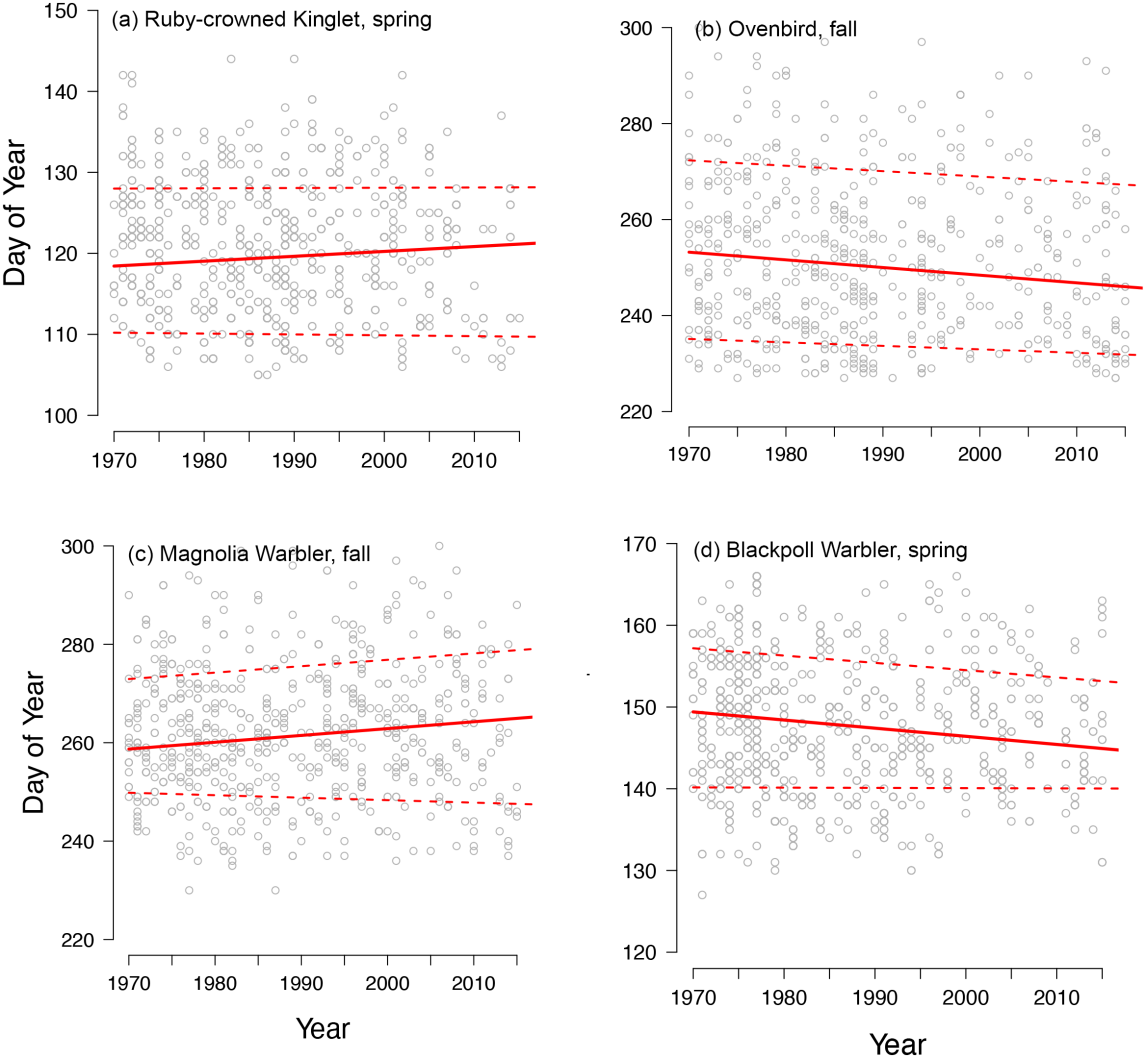
Diverse phenological responses occurred within songbird migrants from 1970 to 2015 at Manomet. We observed all four possible changes in passage duration as outlined in Fig. 1: (A) no change, (B) parallel change, (C) increase and (D) decrease in passage duration. Points shown are first capture dates of four passerine species in either spring or fall at Manomet from 1970 to 2015. Solid line indicates median (0.5 quantile), and lower and upper dotted lines indicate 0.15 and 0.85 quantiles, respectively, fit using quantile regression. Sample sizes are listed in Table 1.

Spring migration was characterized by parallel responses across the distribution of arrival dates, especially for short- and medium-distance migrants, leading to an average increase in passage duration of 0.07 days per decade over the study period (Figs. 2, 4A and 4B). Although eight species exhibited parallel changes in phenology, we found three significant changes in passage duration in spring: two were contractions (blackpoll warbler: −0.88 days per decade, SE = 0.33; magnolia warbler: −0.48 days per decade, SE = 0.22), while the third was an expansion (Swainson’s thrush: +0.138 days per decade, SE = 0.31). Non-parallel responses in spring were not consistently driven by a change in early-arriving birds (Fig. 2).

In contrast, fall migration was characterized by heterogeneous shifts in migration timing among quantiles (Fig. 3). Across all species, passage duration increased on average by 0.45 days per decade, with significant increases in passage duration for five species and no significant decreases (Figs. 3D and 4C). These increases in passage duration were driven by strong delays in the arrival timing of the latest arriving birds (0.85 quantile, Fig. 3). In fact, there were delays in the 0.85 quantile for most species, with short-distance migrants exhibiting the strongest delays. Though two medium-distance migrants had the strongest non-parallel responses in our study (magnolia warbler: +1.61 days per decade; black-and-white warbler: +3.04 days per decade), the other medium-distance migrant showed negligible change in passage duration (Fig. 3D).

Across both seasons, we found just three significant decreases in stopover duration across both seasons (spring blackpoll warbler: slope = −0.04 days per decade, *F* = 5.28, *p* = 0.026; fall Swainson’s thrush: slope = −0.14 days per decade, *F* = 5.23, *p* = 0.027; fall black-and-white warbler: slope = −0.02 days per decade, *F* = 9.47, *p* = 0.004). Three other species in spring showed a non-significant trend towards shorter stopover (Table 2, *p* < 0.1, Swainson’s thrush, black-and-white warbler, hermit thrush). There were no obvious patterns across seasons or migration distances in which species exhibited changes in stopover duration, though two species exhibited a change or a trend in stopover duration in both seasons. Although we did not find widespread systematic changes in stopover duration, we found strong variation in stopover duration among years for some species but not others, as evidenced by the variance terms in our hierarchical model (range: 0.006–0.355, Table S1).

**Figure 5:**
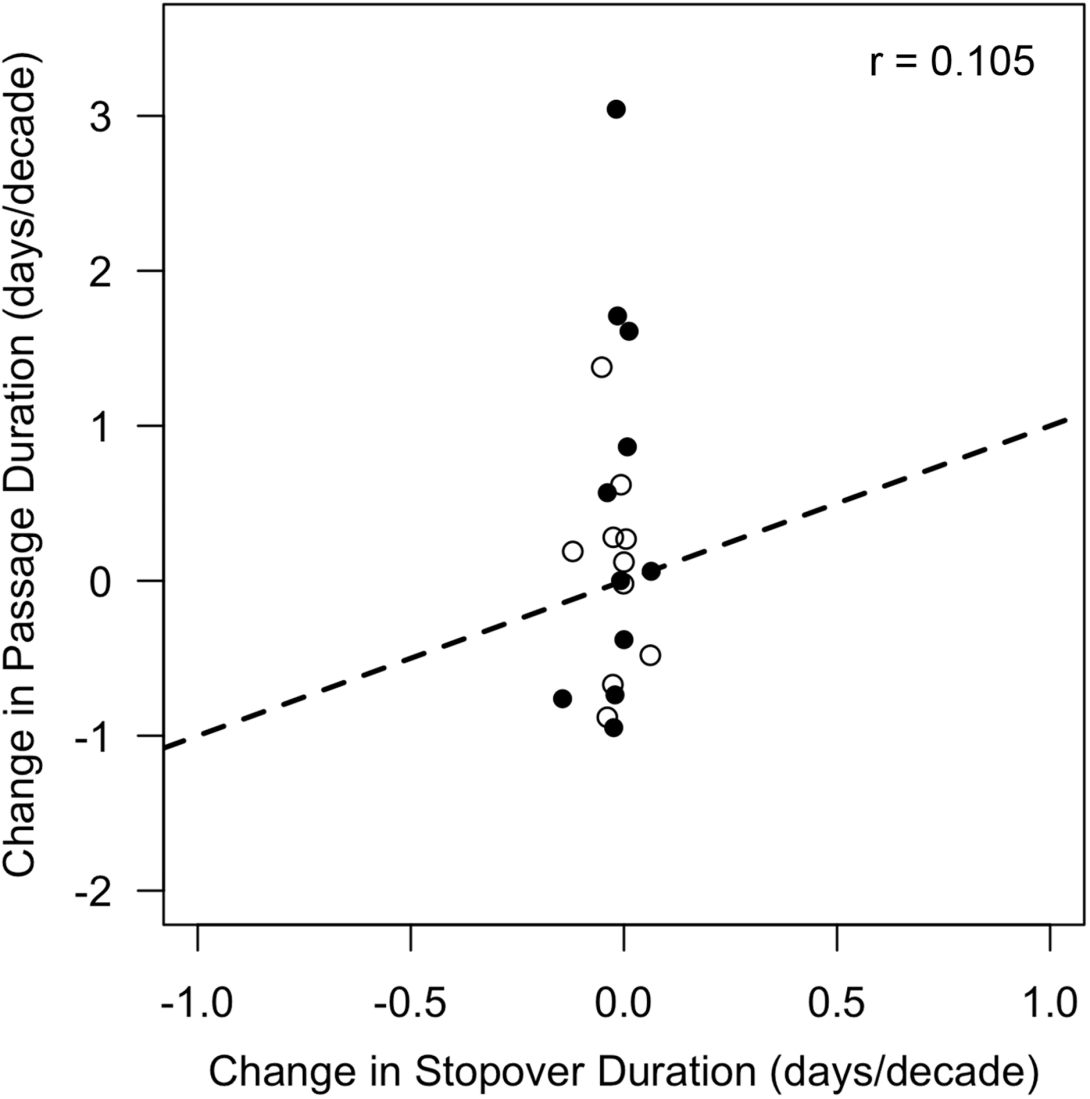
Changes in stopover duration were not correlated with changes in passage duration. Data shown are rates of change in passage duration calculated using quantile regression (0.85–0.15 quantile) vs. rates of change in stopover duration calculated via linear regression of stopover duration estimates over time. Each point represents a single species, with open circles indicating rates calculated from spring migration data, and closed circles indicating those calculated from fall migration data. Dotted line represents a 1:1 relationship (note axis limits), which would be satisfied if changes in stopover duration from 1970 to 2015 were sufficient in magnitude and direction to explain changes in passage duration. Sample sizes are listed in Table 1.

Thus, changes in stopover duration were insufficient to explain corresponding shifts in passage duration (*r* = 0.105, *p* = 0.659) because either increases in passage duration were accompanied by decreases in stopover duration (e.g., fall black-and-white warbler), or the magnitude of change in stopover duration was much less (5.3–22.6 times less) than the magnitude of asymmetry (Fig. 5).

## Discussion

It is widely accepted that the migration timing of songbirds is changing (Parmesan, 2007). Many studies focus solely on changes in the mean date of timing, which implicitly assume parallel changes through time by the earliest- and the latest-arriving birds. Yet, this assumption is not always true. Using a continuous 46-year dataset of bird migration from Manomet, in MA, USA, we documented non-parallel shifts in migration timing, which led to increases in fall, but not spring passage duration. However, changes in stopover duration over the same period could not explain our findings. Our study demonstrates the importance of considering phenological responses across the entire distribution and that the mechanisms underlying systematic changes in phenology are likely complex and species-specific.

We found that the median date of spring migration at Manomet was advancing by 1.04 days per decade, consistent with past studies analyzing both median and mean arrival date of neotropical and trans-Saharan migrants (Marra et al., 2005; Jonzén et al., 2006; Cadahía et al., 2017; Mayor et al., 2017). Phenological advances in spring were parallel across the distribution of arrival dates, especially for short- and medium-distance migrants. This means that the passage duration of spring migration at Manomet has not changed substantially over the past 46 years (on average +0.07 days per decade). This result challenges findings from other studies that found non-parallel responses in spring passage duration migration created by strong advances in early-arriving, but not late-arriving birds (Van Buskirk, Mulvihill & Leberman, 2009; Van Buskirk, 2012; Horton et al., 2019; Lehikoinen et al., 2019). Since we observed a parallel response in spring, it could mean that birds within a migratory population experience homogeneous environmental cues on their wintering grounds that they use to initiate migration, such as warming temperatures (Miller-Rushing et al., 2008) or precipitation (Studds & Marra, 2011). Alternatively, symmetry in migration timing could be due to selection for earlier migration timing across an entire species’ range (Pulido et al., 2001).

Interestingly, non-parallel responses in spring, although weak on average, were strong in long-distance migrants (Fig. 3D): Swainson’s thrush increased its passage duration by 6.4 days over the course of the study and blackpoll warbler decreased its passage duration by 4 days. As has been found before, the median rate of advancement of long-distance migrants in spring was comparable to migrants traveling shorter distances (Jonzén et al., 2006). Taken together, these two findings support recent claims that environmental cues—once thought to be used by short-distance, but not long-distance migrants—may also be important for migration timing in long-distance migrants (Van Buskirk, Mulvihill & Leberman, 2009; Studds & Marra, 2011; Cohen, Moore & Fischer, 2012).

Changes in fall migration phenology have received considerably less attention in the literature, though they are generally less consistent than changes in spring (Thorup, Tøttrup & Rahbek, 2007; Gallinat, Primack & Wagner, 2015). Across species, we found varied changes in fall median arrival date, though most of the significant shifts were delays (Fig. 3B). An average delay of 0.80 days per decade is similar to estimates for changes in songbird migration timing from elsewhere in North America and Europe, and could indicate that the start of migration is delayed due to a later onset of fall conditions or a protraction of the breeding season (range: −0.04 to +3.0 days per decade, Tøttrup, Thorup & Rahbek, 2006; Smith & Paton, 2011; Barton & Sandercock, 2018). Notably, we found increases in fall passage duration of 0.45 days per decade driven by delays in the latest-arriving birds (Fig. 3). Accordingly, this finding corroborates studies that also found non-parallel responses in fall (Van Buskirk, Mulvihill & Leberman, 2009; Barton & Sandercock, 2018; Covino, Horton & Morris, 2020), but not with those that found parallel responses (Mills, 2005; Tottrup, Thorup & Rahbek, 2006), illustrating that changes in fall migration timing remain hard to generalize across time, space and species.

To explain these findings, we tested whether changes in stopover duration were sufficient to explain the observed changes in passage duration. In line with past studies, however, we found little evidence of substantial long-term changes in songbird stopover duration (Hochachka & Fiedler, 2008; Calvert, Taylor & Walde, 2009; Calvert et al., 2012). Consequently, changes in stopover duration could not explain non-parallel responses in phenology (Fig. 5). The three significant changes in stopover duration that we did find were all negative, with three additional species also exhibiting negative trends, averaging 0.066 fewer stopover days per decade. Since this change amounts to less than a third of a day over the entire study period, it suggests that, selective pressures aside (Kokko, 1999), stopover duration for songbirds is relatively inflexible and not as sensitive to environmental change as is migration timing. Still, the shorter lengths of stay that we observed could mean that birds leave the site thin due to pressure to reach breeding grounds (Rakhimberdiev et al., 2018) or that changes in resource availability lead to low refueling rates (Schaub, Jenni & Bairlein, 2008). For example, the strong decreases in fall stopover duration of Swainson’s thrush could be the result of widespread insect declines (Hallmann et al., 2017) or lack of preferred fruits (Gallinat, Primack & Lloyd-Evans, 2020) combined with pressure to reach wintering grounds (Ruiz-Gutierrez et al., 2016). Systematic changes in stopover duration likely result from the complex interaction between selective pressures, heterogeneity in fueling rates, and environmental change (Moore, 2018).

Since changes in stopover duration could not explain changes in passage duration, we hypothesize that three other ecological factors could be generating the observed non-parallel responses: (1) changes in population age-structure, (2) cryptic geographic variation within migratory populations and (3) changing resource availability.

First, changes in the age-structure within a population of migrants could generate the observed increase in fall passage duration. If some age classes always depart later from breeding grounds or are more variable in their departure dates than others, this could lead to an increased variance in arrival dates, and consequently, an increase in passage duration. For some bird species, immatures have a more variable departure timing than adults (Battley, 2006). Subsequent analysis of our data indicates that some, but not all, species had significant changes in the proportion of hatch-years passing through the site in fall between 1970 and 2015 (Table S2). For example, we observed a strong increase in the proportion of black-and-white warbler hatch-years arriving at Manomet over the course of our study, which could explain its strong increase in passage duration (+3.04 days/decade; Table S2; Fig. 3D). We did not find a change in age-structure for brown creeper or magnolia warbler, however, and they also exhibited non-parallel responses, so this explanation is likely species-specific (Table S2).

Second, individuals caught at Manomet could be sourced from multiple geographically distinct populations. Often, birds exhibit weak migratory connectivity, in which individuals from disparate breeding or wintering populations mix at stopover sites during migration (Webster et al., 2002; Ruegg et al., 2014; Trierweiler et al., 2014). If spatiotemporal variation in warming delays historically late, but not early subpopulations from departing breeding grounds, an asymmetry in migration timing between early and late arriving birds could occur (Marra et al., 2005). There is some evidence from analysis of stable isotopes that blackpoll warblers passing through Manomet are sourced from multiple populations (Holberton et al., 2015), though the extent to which changes in the environment affect their departure from breeding grounds is unknown.

Last, changes in fall resource availability could increase passage duration. Across the northeast USA, landscape-scale changes in vegetation have taken place, with fruiting shrub communities shifting from native to invasive plants (Ehrenfeld, 1997). It remains unclear, however, whether migratory songbirds prefer to feed on native or invasive fruits during autumn stopover. Invasive fruits have been found to be nutritionally inferior to native fruits (Smith, DeSando & Pagano, 2013), so adequately refueling with invasive fruits requires more time. If birds prefer invasive fruits (LaFleur, Rubega & Elphick, 2007), a higher abundance and later fruiting phenology of invasive plants may have led birds to spend more time refueling north of Manomet. Alternatively, if birds prefer native fruits (Gallinat, Primack & Lloyd-Evans, 2020), then increased heterogeneity in native fruit availability may have increased heterogeneity in search time and decreased refueling rates within a population of migrants. Low refueling rates are associated with shorter stopover durations in songbird migrants (Yong & Moore, 1993; Schaub, Jenni & Bairlein, 2008), though variation among individuals may mask underlying changes in stopover duration (Lok et al., 2019). Either of these resource-driven mechanisms could generate the increase in passage duration we found in fall. In the future, more extensive datasets on landscape-level phenology of native and invasive plants (e.g., National Phenology Network), and knowledge of fine-scale movements of birds across the stopover landscape will help to contextualize our findings (Smetzer & King, 2018).

Across studies, variation in change in migration timing is prevalent (Table 6 in Barton & Sandercock, 2018: SD rates of spring change = 1.14 days per decade; SD rates of fall change = 1.09 days per decade). This variation can partly be attributed to differences in the spatial and temporal scale of the observations. Many studies that analyze phenological change in birds use banding data from just a single stopover site, making it hard to know whether findings hold for other sites throughout the migratory journey of a species (e.g., this study, Miller-Rushing et al., 2008, but see Gordo & Sanz, 2006; Horton et al., 2019; Lehikoinen et al., 2019; Covino, Horton & Morris, 2020). And even for studies conducted at the same site, conclusions can vary. Phenological change in spring was greater in our study than reported by Miller-Rushing et al. (2008), who analyzed changes in mean arrival date at Manomet using a dataset 13 years shorter than ours. Two-thirds of the species that were analyzed in both studies exhibited stronger advances in the same direction our study (Fig. 2B), consistent with trends in other taxa that rates of phenological change have accelerated in recent years (Bartomeus et al., 2013; Post, Steinman & Mann, 2018). Second, comparing our study with that of Van Buskirk, Mulvihill & Leberman (2009) illustrates how findings differ across space even for datasets of equal length: using five decades of bird banding data, they found non-parallel responses in spring duration driven by strong phenological advances in early arriving birds, whereas we found parallel changes in spring. Resolving these discrepancies will require a unified analysis of regional phenological trends. Hierarchical models that share data among sites are a logical solution to this problem.

## Conclusions

Over the past five decades, ornithologists, ecologists, and natural historians have documented change in the phenology and nature of animal migration (Cotton, 2003; Visser et al., 2009). Birds have received substantial attention, yet most estimates are limited in scope because they assume that changes are parallel across the distribution of arrival times. Using a long-term bird banding dataset, we demonstrate that changes in arrival date at a migratory stopover site can differ between early- and late-arriving individuals, leading to significant increases and decreases in the total time that species is observed. We found that more species exhibited changes in passage duration in fall than in spring demonstrating that the responses of songbirds to environmental change varies within and among species. Our study raises more questions than it answers by suggesting that, contrary to recent predictions, changes in stopover duration do not generate changes in songbird migration phenology (Horton et al., 2020). We conclude that no single factor is most important for changes in migration timing; rather they stem from the interaction of diverse intrinsic and extrinsic factors. Now more than ever, developing an unbiased and nuanced understanding of migration is paramount to the conservation of declining bird (Kamm et al., 2019), insect (Schultz et al., 2017) and mammal migrants (Harris et al., 2009). We hope this work inspires future studies to consider changes in migration timing as the complex and dynamic process that it is.

## Supplemental Information

10.7717/peerj.8975/supp-1 Supplemental Information 1 Supplementary Materials. Includes code used to run quantile regression and Bayesian models, results of stopover duration analyses, and supplemental figures.

10.7717/peerj.8975/supp-2 Supplemental Information 2 Code used to evaluate changes in migration timing across the distribution of arrival dates.

10.7717/peerj.8975/supp-3 Supplemental Information 3 Code used to create capture histories from banding data provided on data repository.

10.7717/peerj.8975/supp-4 Supplemental Information 4 Code used to run hierarchical mark-recapture models to estimate annual stopover duration.

## Additional Information and Declarations

### Data Availability

The following information was supplied regarding data availability: Data is available on Open Science Framework: Dorian, Nicholas, Trevor Lloyd-Evans, and J.M. Reed. 2019. “Can Changes in Songbird Stopover Duration Explain Observed Shifts in Migration Phenology?” OSF. December 20. DOI 10.17605/OSF.IO/QV7Z9.
