# Supplementary material for: Non-parallel changes in songbird migration timing are not explained by changes in stopover duration
Source: PeerJ. 2020 May 19;8:e8975. doi: 10.7717/peerj.8975 (PMC7243817; doi:10.7717/peerj.8975)
Supplement: Supplemental Information 1 — Includes code used to run quantile regression and Bayesian models, results of stopover duration analyses, and supplemental figures. [file peerj-08-8975-s001.docx]

**Electronic Supplementary Materials**

Asymmetric shifts in fall songbird migration phenology are not explained by changes in stopover duration

Nicholas N. Dorian, Trevor Lloyd-Evans, and J. Michael Reed

Last revised: December 17, 2019

**Table of Contents**

**Abbreviations**……………………………………………………………...……….…….…….1

**1**…………………………………………………………………….R Code, uploaded separately

Code to conduct quantile regression

**2A**…………………………………………………………………...R Code, uploaded separately

Code to generate capture histories for CJS analysis **2B**……………………………………………………………………………………………..…2

Model code for hierarchical CJS models **2C**…………………………………………………………………...R Code, uploaded separately

Script used to run CJS models using JAGS 4.0 in R.

**2D**…………………………………………………………………………………………….…3

Functions from Kéry and Schaub 2012 needed to run Bayesian state-space CJS and MSMR models.

**3, Table S1**……………………………………………………………………………………...4

Median standard deviation (σ) and 95% credible interval of hierarchically distributed parameters, survival (φ) and recapture (*p*) probabilities, and their variances.

**4**…………………………………………………………………………………………………6

Trends in stopover duration over time for each species

**5, Table S2**……………………………………………………………………………….….....17

Changes in the proportion of hatch-year birds passing through Manomet in fall

**6. References**…………………………………………………………………………………..17

**Abbreviations**

VEER: Veery *Catharus fuscescens*

SWTH: Swainson’s Thrush *Catharus ustulatus*

BLPW: Blackpoll Warbler *Setophaga striata*

CAWA: Canada Warbler *Cardellina canadensis*

OVEN: Ovenbird *Seiurus aurocapilla*

BAWW: Black-and-white Warbler *Mniotilta varia*

MAWA: Magnolia Warbler *Setophaga magnolia*

HETH: Hermit Thrush *Catharus guttatus*

SWSP: Swamp Sparrow *Melospiza georgiana*

BRCR: Brown Creeper *Certhia americana*

RCKI: Ruby-crowned Kinglet *Regulus calendula*

CJS: Cormack-Jolly-Seber mark-recapture model

QR: Quantile regression

**Supplementary Material 2A.** Model code for all hierarchical group-effects CJS models. Models for all species in both spring and fall were run across three chains for 30,000 iterations each with a burn-in of 10,000 iterations, retaining one third of all draws. There were insufficient recapture data for Brown Creeper in spring to reach convergence, so that dataset was omitted from future analyses. Adapted from Kéry and Schaub 2012, ch. 7. Last revised on December 17th, 2019 by: N.N.Dorian

model{

sigma.phi~dunif(0,5) #prior on survival random effects

tau.phi<-pow(sigma.phi, -2)#calculate precision: 1/variance

sigma2.phi<-pow(sigma.phi,2)#variance

sigma.p~dunif(0,5)#prior on recapture random effects

tau.p<-pow(sigma.p, -2)#calculate precision: 1/variance

sigma2.p<-pow(sigma.p,2)#variance

phi.logit~dnorm(1,0.001) #prior for survival on logit-scale

phi.mu<-1/(1+exp(-phi.logit)) #back-transform grand mean survival to real scale

stay.mu<-(-1/log(phi.mu)) #calculate stopover duration

p.logit~dnorm(0,0.001) #prior for recapture on logit-scale

p.mu<-1/(1+exp(-p.logit)) #back-transform recapture to real scale

for(i in 1:nyear){ #loop over years to generate random effects prior for each year

year.phi[i]~dnorm(phi.logit,tau.phi) #mean-zero normal prior, variance term = precision

year.p[i]~dnorm(p.logit,tau.p)#mean-zero normal prior, variance term = precision

}

#set priors for each individual based on group and year

for(i in 1:nind){

for(t in f[i]:(n.occasions-1)){

logit(phi[i,t])<-year.phi[year[i]]

logit(p[i,t])<-year.p[year[i]]

}

}

#likelihood

for(i in 1:nind){

z[i, f[i]]<-1

for(t in (f[i]+1):n.occasions){

#state process

mu1[i,t]<-phi[i,t-1]*z[i,t-1]

z[i,t]~dbern(mu1[i,t])

#observation process

mu2[i,t]<-p[i,t-1]*z[i,t]

y[i,t]~dbern(mu2[i,t])

}

}

}

**Supplementary Material 2D.** Functions from Kéry and Schaub 2012 needed to run Bayesian state-space CJS. Models using JAGS 4.0 to estimate stopover duration of migrant songbirds

This script contains 3 functions: get.first to retrieve the date of first capture for each individual, and two functions that generate initial values needed to improve convergence.

This code is necessary to run the sample R code in files S1C and S2C.

Author: Kéry, M. and M. Schaub. 2012. Bayesian Population Analysis using WinBUGS: A hierarchical perspective. Chps 7 & 9. Academic Press, Cambridge.

Last revised: January 10th, 2019

Function for both models

This function retrieves the column (sampling occasion) of the first capture. When used with apply() in the sample R code, it iterates over all individuals.

get.first<-function(x)min(which(x!=0))

CJS-specific functions

#This function takes a capture history matrix (rows = individuals, columns = sampling occasions) to and returns a matrix with "known" information. For example, the capture history 010010 is converted into 011110 because we know that the individual was alive in between the two capture occasions).

known.states.cjs<-function(ch){

state<-ch

for(i in 1:dim(ch)[1]){

n1<-min(which(ch[i,]==1))

n2<-max(which(ch[i,]==1))

state[i,n1:n2]<-1

state[i,n1]<-NA

}

state[state == 0]<-NA

return(state)

}

#This function generates initial values for each individual and turns the rest of the capture history into NAs. This function is used to improve model convergence as well.

cjs.init.z<-function(ch,f){

for(i in 1:dim(ch)[1]){

if(sum(ch[i,])==1)next

n2<-max(which(ch[i,]==1))

ch[i,f[i]:n2]<-NA

}

for(i in 1:dim(ch)[1]){

ch[i,1:f[i]]<-NA

}

return(ch)

}

**Supplementary Material 3.**

**Table S1** Median variance (σ^2^) and 95% credible interval of hierarchically distributed parameters, survival and recapture probabilities. Stopover duration estimates are presented in the text under the transformation -1/log(survival) (Schaub et al., 2001). Random effects terms of among-year variance in survival and recapture probabilities are presented on the logit-scale of the parameter. Hierarchical models were run for each species-season combination using CJS models with a random effect of year. Description of this model is provided in the main text. As expected, sparse datasets yield poor estimates of σ^2^ (wide credible intervals, i.e. veery spring), whereas rich datasets have more precise estimates.

| **Species** | **Parameter** | *n* | **Spring** | *n* | **Fall** |
| --- | --- | --- | --- | --- | --- |
| VEER | Survival | 610 | 0.524 (0.441-0.584) | 542 | 0.767 (0.717-0.798) |
| VEER | Survival σ^2^ |  | 0.0393 (0.000-0.356) |  | 0.050 (0.000-0.294) |
| VEER | Recapture |  | 0.148 (0.091-0.221) |  | 0.134 (0.108-0.157) |
| VEER | Recapture σ^2^ |  | 0.742 (0.138-2.19) |  | 0.018 (0.000-0.214) |
| SWTH | Survival | 1505 | 0.417 (0.304-0.517) | 1233 | 0.751 (0.697-0.793) |
| SWTH | Survival σ^2^ |  | 0.297 (0.063-0.939) |  | 0.109 (0.007-0.332) |
| SWTH | Recapture |  | 0.075 (0.046-0.116) |  | 0.086 (0.062-0.106) |
| SWTH | Recapture σ^2^ |  | 0.281 (0.002-1.33) |  | 0.109 (0.001-0.484) |
| BLPW | Survival | 1101 | 0.427 (0.088-0.629) | 7512 | 0.713 (0.659-0.743) |
| BLPW | Survival σ^2^ |  | 0.355 (0.000-3.95) |  | 0.082 (0.019-0.336) |
| BLPW | Recapture |  | 0.012 (0.002-0.044) |  | 0.026 (0.017-0.038) |
| BLPW | Recapture σ^2^ |  | 1.01 (0.016-6.38) |  | 1.18 (0.612-2.27) |
| CAWA | Survival | 1452 | 0.545 (0.458-0.609) | 467 | 0.731 (0.677-0.769) |
| CAWA | Survival σ^2^ |  | 0.085 (0.000-0.409) |  | 0.042 (0.000-0.301) |
| CAWA | Recapture |  | 0.076 (0.050-0.106) |  | 0.14 (0.103-0.178) |
| CAWA | Recapture σ^2^ |  | 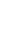0.227 (0.005-0.886) |  | 0.14 (0.001-0.660) |
| OVEN | Survival | 1488 | 0.623 (0.559-0.670) | 651 | 0.806 (0.775-0.827) |
| OVEN | Survival σ^2^ |  | 0.102 (0.004-0.364) |  | 0.019 (0.000-0.136) |
| OVEN | Recapture |  | 0.099 (0.074-0.129) |  | 0.097 (0.080-0.117) |
| OVEN | Recapture σ^2^ |  | 0.266 (0.076-0.666) |  | 0.0241 (0.000-0.191) |
| BAWW | Survival | 3351 | 0.658 (0.626-0.684) | 793 | 0.759 (0.733-0.783) |
| BAWW | Survival σ^2^ |  | 0.023 (0.000-0.089) |  | 0.006 (0.000-0.068) |
| BAWW | Recapture |  | 0.114 (0.096-0.133) |  | 0.101 (0.081-0.122) |
| BAWW | Recapture σ^2^ |  | 0.074 (0.002-0.232) |  | 0.158 (0.054-0.369) |
| MAWA | Survival | 2423 | 0.577 (0.512-0.633) | 1079 | 0.687 (0.638-0.731) |
| MAWA | Survival σ^2^ |  | 0.197 (0.047-0.51) |  | 0.032 (0.000-0.190) |
| MAWA | Recapture |  | 0.054 (0.042-0.068) |  | 0.084 (0.064-0.109) |
| MAWA | Recapture σ^2^ |  | 0.133 (0.007-0.446) |  | 0.043 (0.001-0.264) |
| HETH | Survival | 1540 | 0.714 (0.674-0.746) | 1929 | 0.798 (0.782-0.812) |
| HETH | Survival σ^2^ |  | 0.130 (0.044-0.317) |  | 0.0364 (0.006-0.097) |
| HETH | Recapture |  | 0.162 (0.131-0.193) |  | 0.187 (0.167-0.207) |
| HETH | Recapture σ^2^ |  | 0.254 (0.107-0.542) |  | 0.111 (0.054-0.219) |
| SWSP | Survival | 1327 | 0.701 (0.661-0.731) | 1126 | 0.794 (0.752-0.821) |
| SWSP | Survival σ^2^ |  | 0.035 (0.000-0.189) |  | 0.067 (0.048-0.088) |
| SWSP | Recapture |  | 0.102 (0.079-0.127) |  | 0.116 (0.002-0.403) |
| SWSP | Recapture σ^2^ |  | 0.256 (0.085-0.604) |  | 0.549 (0.274-1.08) |
| BRCR | Survival | 187 | INSUFFICIENT DATA | 1587 | 0.573 (0.521-0.616) |
| BRCR | Survival σ^2^ |  | INSUFFICIENT DATA |  | 0.059 (0.001-0.242) |
| BRCR | Recapture |  | INSUFFICIENT DATA |  | 0.164 (0.130-0.200) |
| BRCR | Recapture σ^2^ |  | INSUFFICIENT DATA |  | 0.162 (0.013-0.463) |
| RCKI | Survival | 1235 | 0.635 (0.569-0.684) | 1848 | 0.596 (0.541-0.643) |
| RCKI | Survival σ^2^ |  | 0.024 (0.000-0.285) |  | 0.142 (0.025-0.349) |
| RCKI | Recapture |  | 0.090 (0.062-0.123) |  | 0.089 (0.072-0.109) |
| RCKI | Recapture σ^2^ |  | 0.459 (0.126-1.10) |  | 0.027 (0.000-0.213) |

**Supplementary Material 4.**

Trends in stopover duration over time for each species in each season at Manomet between 1970 and 2015. Slope values are presented in main text. Points represent median stopover duration estimates from CJS models +/- 95% credible intervals. Red lines indicate linear regressions fit to these data. Slope values and significance of trend lines are reported in Table 2 in the main text.

VEER Fall

**Supplementary Material 5.**

One way that the variance in arrival dates (i.e. increase in migration duration) could increase at Manomet is via a change in the age-distribution of birds passing through Manomet. Since different ages may initiate migration at different times, as has been found before for some species of birds (Battley, 2006), if the age distribution has changed of the sampled population, this would be sufficient to generate asymmetries in migration timing. To that end, we analyzed trends in the proportion of hatch-year birds passing through Manomet of each species. We used beta regression with a logit-link (implemented via the package *betareg* in R) because our data were proportions bound between 0 and 1. Significance was assessed using a type II marginal hypothesis test implemented via the Anova() function in the *car* package in R. We found significant increases in the proportion of hatch-years captured for Swainson’s Thrush, Black-and-white Warbler, and Hermit Thrush.

**Table S2** Changes in the proportion of hatch-year birds passing through Manomet in fall from 1970–2015. Three species showed significant increases in the proportion of hatch-years captured at the site, which could be a factor that generated our observed increase in variance of arrival date. Slopes were calculated using beta regression and are on reported on the logit scale. Bolded p-values indicate significance at the 0.05 level.

| Species | Slope | SE | X^2^ | *p* |
| --- | --- | --- | --- | --- |
| VEER | 0.001 | 0.003 | 0.009 | 0.72 |
| SWTH | 0.034 | 0.12 | 8.65 | **0.003** |
| BLPW | 0.012 | 0.007 | 2.53 | 0.112 |
| CAWA | 0.783 | 0.011 | 0.783 | 0.376 |
| OVEN | -0.010 | 0.012 | 0.685 | 0.408 |
| BAWW | 0.264 | 0.011 | 5.36 | **0.021** |
| MAWA | -0.0013 | 0.012 | 0.012 | 0.913 |
| HETH | 0.032 | 0.010 | 9.81 | **0.0017** |
| SWSP | 0.0177 | 0.011 | 2.46 | 0.117 |
| BRCR | -0.011 | 0.007 | 2.71 | 0.099 |
| RCKI | -0.012 | 0.007 | 3.33 | 0.068 |

**Supplementary Material 6.**

**References:**

Battley PF. 2006. Consistent annual schedules in a migratory shorebird. *Biology Letters* 2:517–520. DOI: 10.1098/rsbl.2006.0535.

Kéry M, Schaub M. 2012. *Bayesian population analysis using WinBUGS: a hierarchical perspective*. Cambridge: Academic Press.

Schaub M, Pradel R, Jenni L, Lebreton J, Ecology S, Mar N. 2001. Migrating birds stop over longer than usually thought: an improved capture-recapture analysis. *Ecology* 82:852–859.
